# Factors influencing the response to periodontal therapy in patients with diabetes: *post hoc* analysis of a randomized clinical trial using machine learning

**DOI:** 10.1590/1678-7757-2025-0211

**Published:** 2025-07-25

**Authors:** Nidia CASTRO DOS SANTOS, Arthur MANGUSSI, Tiago RIBEIRO, Rafael Nascimento de Brito SILVA, Mauro Pedrine SANTAMARIA, Magda FERES, Thomas VAN DYKE, Ana Carolina LORENA

**Affiliations:** 1 Hospital Israelita Albert Einstein Faculdade Israelita de Ciências da Saúde Albert Einstein São Paulo SP Brasil Hospital Israelita Albert Einstein, Faculdade Israelita de Ciências da Saúde Albert Einstein, São Paulo, SP, Brasil.; 2 Universidade Guarulhos Divisão de Pesquisa Odontológica Guarulhos SP Brasil Universidade Guarulhos, Divisão de Pesquisa Odontológica, Guarulhos, SP, Brasil.; 3 ADA Forsyth Institute Cambridge MA United States ADA Forsyth Institute, Cambridge, MA, United States.; 4 Instituto Tecnológico de Aeronáutica Divisão de Ciência da Computação São José dos Campos SP Brasil Instituto Tecnológico de Aeronáutica (ITA), Divisão de Ciência da Computação, São José dos Campos, SP, Brasil.; 5 Universidade Estadual Paulista Instituto de Ciência e Tecnologia Departamento de Diagnóstico e Cirurgia São José dos Campos SP Brasil Universidade Estadual Paulista (UNESP), Instituto de Ciência e Tecnologia, Departamento de Diagnóstico e Cirurgia, São José dos Campos, SP, Brasil.; 6 University of Kentucky College of Dentistry Lexington KY United States University of Kentucky, College of Dentistry, Lexington, KY, United States.; 7 Harvard School of Dental Medicine Department of Oral Medicine, Infection, and Immunity Boston MA United States Harvard School of Dental Medicine, Department of Oral Medicine, Infection, and Immunity, Boston, MA, United States.

**Keywords:** Artificial intelligence, Clinical trial, Diabetes mellitus, Non-surgical periodontal debridement, Periodontitis

## Abstract

**Objective:**

To evaluate factors influencing the response to periodontal therapy in patients with periodontitis and type 2 diabetes mellitus (DM) using machine learning (ML) techniques, considering periodontal parameters, metabolic status, and demographic characteristics.

**Methodology:**

We applied machine learning techniques to perform a post hoc analysis of data collected at baseline and a 6-month follow-up from a randomized clinical trial (RCT). A leave-one-out cross-validation strategy was used for model training and evaluation. We tested seven different algorithms: K-Nearest Neighbors, Decision Tree, Support Vector Machine, Random Forest, Extreme Gradient Boosting, and Logistic Regression. Model performance was assessed using accuracy, specificity, recall, and the area under the Receiver Operating Characteristic (ROC) curve (AUC).

**Results:**

a total of 75 patients were included. Using the first exploratory data analysis, we observed three clusters of patients who achieved the clinical endpoint related to HbA1c values. HbA1c ≤ 9.4% was correlated with lower PD (r=0.2), CAL (r=0.1), and the number of sites with PD ≥5 mm (r=0.1) at baseline. This study induced AI classification models with different biases. The model with the best fit was Random Forest with a 0.83 AUC. The Random Forest AI model has an accuracy of 80%, a sensitivity of 64%, and a specificity of 87%. Our findings demonstrate that PD and CAL were the most important variables contributing to the predictive performance of the Random Forest model.

**Conclusion:**

The combination of nine baseline periodontal, metabolic, and demographic factors from patients with periodontitis and type 2 DM may indicate the response to periodontal therapy. Lower levels of full mouth PD, CAL, plaque index, and HbA1c at baseline increased the chances of achieving the endpoint for treatment at 6-month follow-up. However, all nine features included in the model should be considered for treatment outcome predictability. Clinicians may consider the characterization of periodontal therapy response to implement personalized care and treatment decision-making. Clinical trial registration ID: NCT02800252

## Graphical abstract

**Figure:**
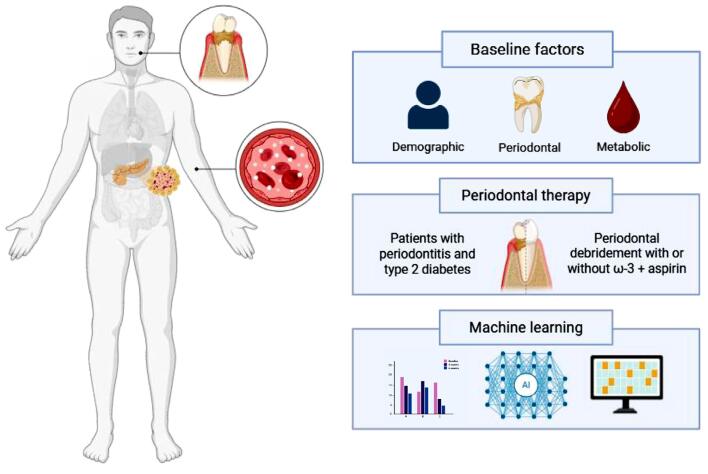

## Introduction

Periodontitis is an inflammatory disease associated with a dysbiotic biofilm characterized by the progressive destruction of the tooth-supporting tissues.^1^ The severe form of periodontitis is the sixth most prevalent condition globally, affecting 11% of the population.^2^ Diabetes mellitus (DM) is a metabolic disorder characterized by chronic hyperglycemia, which predisposes patients to microvascular end-organ complications.^3^ Periodontitis and DM are non-communicable diseases that share a bidirectional relationship—periodontitis impairs glycemic control, while DM increases the prevalence, severity, and progression of periodontitis.^4^ Managing these diseases requires multifactorial behavioral and therapeutic approaches in order to delay complications and increase longevity and quality of life.

Precision dentistry/medicine is based on tailoring therapy to individuals.^5^ In recent decades, our ability to understand human variation using clinical and biological markers, improving data acquisition, analysis, and interpretation, and guiding preventive care and therapeutic interventions based on scientific evidence, has dramatically improved.^3^ Oral health research has focused on association studies to establish risk factors/indicators in health and disease progression via cross-sectional studies.^5^ However, association studies do not enable the inference of prediction values for treatment outcomes. In this regard, datasets from RCTs, particularly those with transparent and reproducible methods, may be useful for building prediction models for treatment outcomes.^6^

AI intends to reproduce the human cognitive process and achieve the same outcome as medical professionals within a much shorter timeframe.^7^ In dentistry, AI has been applied to distinguish between lesions and typical structures, prioritize risk factors, improve diagnosis, as well as stimulate and evaluate prospective results.^7,8^ In periodontics, AI has been used to identify periodontally compromised premolars and molars via radiographs with accuracies of 81% and 76.6%, respectively. Another application was exploring the possible biological differences between the formerly known chronic and aggressive periodontitis using machine learning models, suggesting limited dissimilarities between the two forms of the disease.^9,10^ The personalization of periodontal treatments for patients with type 2 DM based on clinical trial data is limited by the relative homogeneity of the study population and the restricted availability of variables, which may hinder the identification of subgroups with distinct therapeutic responses. In this context, applying machine learning (ML) methods to clinical periodontal data, glycated hemoglobin levels, and demographic characteristics can uncover subtle patterns and interactions that are not easily detectable when using traditional statistical approaches, which contributes to a more refined understanding of the factors that influence treatment outcomes. This approach is particularly relevant given the additional complexity imposed by type 2 DM, which affects inflammatory responses, tissue healing, and infection control, making periodontal treatment outcomes more unpredictable when compared to normoglycemic patients with periodontitis. To date, no studies have evaluated the outcomes of periodontal therapy in patients with DM using RCTs datasets analyzed with AI techniques. Thus, we aimed to evaluate factors that influence the response to periodontal therapy in patients with periodontitis and type 2 DM using ML techniques, considering periodontal parameters, metabolic status, and demographic characteristics at baseline.

## Methodology

### Randomized clinical trial

We performed a *post hoc* analysis of structured data obtained from a placebo-controlled, double-blind, randomized RCT that evaluated the effects of adjunctive dietary supplementation with omega-3 and low-dose aspirin for the treatment of periodontitis in patients with type 2 DM (10) (NCT02800252). Seventy-five patients were included. The RCT was performed from 2016 to 2019. This study was performed in 2023. The trial adhered to the Declaration of Helsinki, and all participants provided written informed consent (CAAE: 51626115.5.0000.0077). The São Paulo State University and the Institute of Science and Technology Ethics Committee approved the study. Periodontal clinical data assessed at baseline and 6 months post-therapy were included in the analysis. The primary outcome, inclusion and exclusion criteria, and clinical protocols have been published before.^11^

### Primary outcome

The primary outcome of this study was a binary variable that assessed whether the patient achieved the clinical endpoint for periodontal treatment or not [≤4 sites with probing depth (PD) ≥5mm] at the last RCT observation.^12^ Patients were classified as “good responders” if they had four or fewer sites with probing depth (PD) ≥5 mm at 6 months. Those with five PD sites or more were considered “poor responders.”

### Inclusion and exclusion criteria

Patients with type 2 diabetes and periodontitis were selected. Eligible participants were adults (≥35 years old) with type 2 diabetes for at least five years, undergoing treatment, and presenting with periodontitis (Stages III/IV, Grades B/C), including at least six sites with PD and attachment loss ≥5 mm, bleeding on probing (BoP), and ≥15 teeth. Exclusion criteria included periodontal therapy in the previous six months; antimicrobial therapy in the previous six months; systemic conditions (other than diabetes) that could affect the progression of periodontitis; long-term use of medication that could interfere with periodontal response; pregnancy or lactation; smoking; and allergies to fish/seafood or aspirin.^11^

### Dataset

According to the inclusion and exclusion criteria, the dataset presents 75 patients with periodontitis and type 2 DM. In addition, it has 31 attributes including endpoint (class), group, gender, age, number of teeth, glycated hemoglobin (HbA1c), cytokines, number and percentage of sites with PD ≤ 3 mm, PD ≥ 4 mm, PD ≥ 5 mm, PD ≥ 6 mm, PD ≥ 7 mm and clinical periodontitis parameters such as PD, clinical attachment level (CAL), BoP, and plaque index (PI). The data for the main clinical parameters of the study at baseline and at the 6-month follow-up were compared using the one-way ANOVA test and the Kruskal-Wallis test.

Regarding patient outcomes, the dataset was imbalanced, with 70.67% of patients not achieving the endpoint. Moreover, 40% of patients present a missing cytokine feature value. Therefore, all cytokine features were excluded, as well as name and group, because they do not contain complete information and could harm prediction performance.

Furthermore, the number and percentage of sites are deemed redundant attributes, leading to the exclusion of the percentage of sites with different PDs. Given the significant relationship between the number of sites and the primary outcome in order to determine whether a patient will achieve the clinical endpoint of treatment, all these features were omitted. After conducting these analyses, the final dataset comprised 75 observations and nine variables: mean full-mouth PD, mean full-mouth CAL, percentage of sites with BoP, percentage of sites with supragingival biofilm (PI), number of teeth, HbA1c levels, use of adjunctive omega-3 and aspirin, age, and gender.

We conducted a descriptive statistical analysis using the dataset, correlating periodontal variables with HbA1c levels at baseline. For this purpose, the mean and standard deviation for each feature were analyzed to better understand the dataset and verify noisy data.

### Model training

K-fold cross-validation is a widely used sampling technique in the ML literature that involves randomly dividing the dataset into K folds of nearly equal sizes.^12^ K-1 folds are used to train the classifier, while the remaining fold is used for testing. This procedure is repeated K times, with each fold serving as the test set once. A particular case of K-fold cross-validation is the leave-one-out (LOO) method, in which K = n is used, with n = 75. This procedure enables us to obtain more precise estimates of classification accuracy for small-sized datasets.^13^

Each training dataset was standardized with a zero mean and unitary variance. Due to the imbalanced nature of the dataset, the SMOTE technique (Synthetic Minority Oversampling Technique) was applied to the training sets. SMOTE works by oversampling the minority class and generating synthetic examples between the nearest neighbors of K within the minority class.^14^

The K-Nearest Neighbors (KNN), Decision Tree (DT), Support Vector Machine (SVM), Random Forest (RF), Extreme Gradient Boost (XGB), Catboost (CB), and Logistic Regression (LR) classification techniques were tested. KNN classifies a test example based on a majority vote of the K nearest neighbors in the training set. DT recursively partitions the training data based on hierarchical tests using the feature values. SVM builds a hyperplane (possibly in a feature space in which non-linearity can be imposed), separating the classes with maximum margin. RF joins the predictions of multiple DTs induced from subsamples of the data. XGB and CB are also ensemble classifiers, joining the predictions of multiple DTs by focusing sequentially on incorrectly classified observations. LR is a statistical method to predict binary outcomes.

The following ML algorithms were tested: KNN, DT, SVM, RF, XGB, CB, and LR. The metrics used to evaluate and compare the performance of each model were accuracy, specificity, and recall. Accuracy is defined as the ratio of correct predictions over the total number of predictions. Specificity gives the true negatives correctly retrieved. Recall, also named sensitivity, refers to the true positives correctly retrieved. Furthermore, Receiver Operating Characteristic (ROC) curves were used to assess the performance of binary classifiers.

Moreover, we analyzed the RF confusion matrix, which is a valuable tool for understanding how the model classifies observations within the dataset. It is a two-dimensional matrix, with the true labels represented on the y-axis and the predicted labels on the x-axis.

For all ML models, a grid search was performed to optimize the hyperparameters of the model for each training fold during all the LOO iterations. The classification models were implemented with the scikit-learn package using Python 3.10.12 in Google Colab. Finally, the function CalibratedClassifierCV from the scikit-learn package was employed to enhance the estimation of the probabilities of classifiers assigned to each class. The requirements to ensure the reproducibility of the results are: joblib==1.3.2, scikit-learn==1.3.0, pandas==2.1.4, numpy==1.26.3, xgboost==2.0.3, catboost==1.2.3, imblearn==0.11.0, and shap==0.45.0.

A SHapley Additive exPlanations (SHAP) unified framework was applied to interpret predictions from complex models.^15^ This framework assigns importance values to each feature for a specific prediction, enhancing the interpretability of ML models. Shapley regression values serve as feature importance specifically designed for linear models dealing with multicollinearity. The process entails retraining the model by including and excluding specific features from the dataset and comparing the predictions in each case. This iterative procedure was performed for all features in the dataset.

## Results

### Exploratory data analysis

A total of 75 patients were included in the analysis [45 female (60%) and 30 male (40%)]. At baseline, the mean age of the population was 55±9 years, presenting a mean of 22±4 teeth and a mean HbA1c of 8.15±1.54 %. For the clinical periodontal parameters, the mean PD was 3.3±0.5 mm, the mean CAL was 3.8±0.8 mm, the mean BoP was 46.6±19.3 %, and the mean PI was 55.3±17.3 %. At six months, the mean HbA1c was 7.9±1.4 % (p=.416), the mean PD was 2.9±0.4 mm (p=.000), the mean CAL was 3.5±0.7 mm (p=.020), the mean BoP was 29.3±14.2 % (p=.000), and the mean PI was 39.6±15.2 % (p=.000). Table 1 shows the results of the comparisons between baseline and six months follow-up.

**Table 1:** Clinical, metabolic, and demographic parameters (mean±SD; median) at baseline and 6 months follow-up.

Parameters	Baseline	6 months	Mean difference	95% C.I. for difference (baseline ; 6 months)	p value
Female (%)	60	..	..	..	..
Age	55±9.2 (56)	..	..	..	..
Number of teeth	21.5±3.9 (22)	..	..	..	..
HbA1c (%)	8.2±1.5 (7.9)	7.9±1.4 (7.8)	..	..	.416
Mean PD (mm)	3.3±0.5 (3.2)	2.9±0.4 (2.8)	-.338±.076	.189;.488	.000
Mean CAL (mm)	3.8±0.8 (3.7)	3.5±0.7 (3.3)	-.290±.123	.046;.534	.020
BoP (%)	46.6±19.3 (48)	29.3±14.2 (31)	-17.335±2.772	11.875;22.813	.000
PI (%)	55.3±17.3 (53.7)	39.6±15.2 (40.7)	-15.648±2.659	10.394;20.903	.000
Number of sites with PD ≥5mm	24.1±16.2 (21)	14.5±11.2 (12)	-9.613±2.273	5.121;14.105	.000

Legends: BoP, bleeding on probing; CAL, clinical attachment level; C.I., confidence interval; HbA1c, glycated hemoglobin; PD, probing depth; PI, plaque index.

For periodontal parameters, intergroup differences were assessed using the One-way ANOVA test (p<.05). HbA1c levels were analyzed using the Kruskal-Wallis test (p<.05).

**Figure 1:**
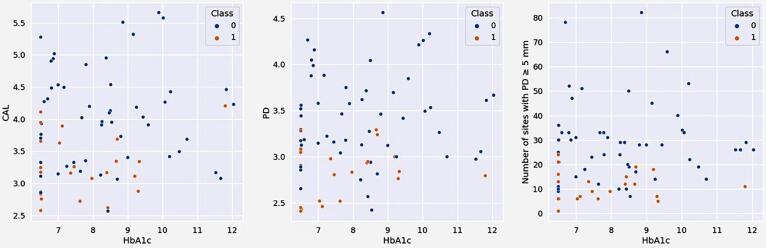
Distribution of patients according to HbA1c levels and periodontal clinical parameters at baseline. The blue dot represents a patient who did not achieve the endpoint for periodontal treatment (≤4 sites with PD≥5 mm) (Class 0), and the orange dot represents a patient who achieved the endpoint for periodontal treatment (≤4 sites with PD≥5 mm) (Class 1).

Of the 75 patients included in the study, 22 achieved the clinical endpoint for periodontal treatment (≤4 sites with PD ≥5 mm) six months post-therapy. Using exploratory data analysis, we observed clusters of patients who achieved the clinical endpoint related to HbA1c values. Of the 22 good responders, 21 (95.45%) presented HbA1c baseline levels ≤ 9.4%, which correlated with lower mean CAL, mean PD, and mean number of sites with PD ≥ 5 mm at baseline. The HbA1c threshold was established based on the dataset employed in this study, using a visual inspection of the distribution of HbA1c values among patients. Figure 1 shows patient distribution according to HbA1c and periodontal clinical parameters at baseline.

### Machine learning model selection and evaluation

**Figure 2:**
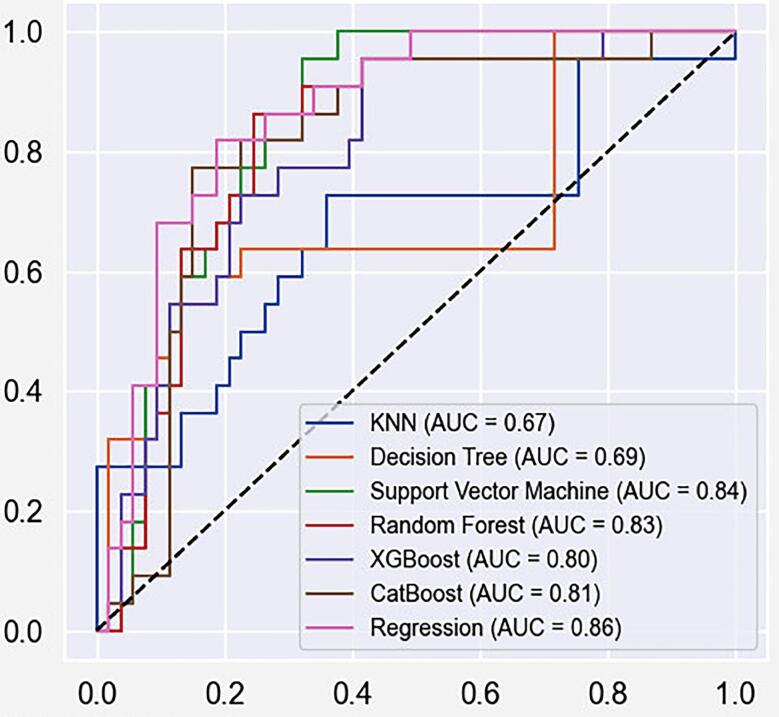
Receiver Operating Characteristic (ROC) curve and Area Under ROC Curve (AUC) values. The ROC Curve for all models tested is displayed, representing the true positive rate versus the false positive rate across a range of cutoffs, along with the AUC for each model tested

In Figure 2, we present the ROC Curve for all models tested, representing the true positive rate versus the false positive rate across a range of cutoffs. In Table 2, we present the metrics for those models. Considering the ROC Curve and the metrics, the RF and LR models achieved the highest accuracy, but RF had higher specificity scores than LR. SVM had similar specificity performance and higher AUC performance than RF. However, the accuracy of SVM was worse than RF. LR has the best recall and AUC scores. The RF and LR models were regarded as the most effective models for our dataset, obtaining a better overall predictive performance regarding the set of metrics considered. However, developing a model to predict treatment response serves the purpose of making the right predictions about true negatives more accurately than positives, justifying the choice of the RF model for subsequent discussions.

**Table 2:** General comparison of evaluation metrics for all classification models induced in this work. The best results per metric are highlighted in bold.

Model	Accuracy	Specificity	Sensitivity	AUC
K-Nearest neighbor	0.680	0.736	0.545	0.671
Decision Tree	0.760	0.849	0.545	0.691
Suppor Vector Machine	0.773	0.868	0.545	0.840
Random Forest	0.800	0.868	0.636	0.835
Extreme Gradient Boosting	0.747	0.811	0.591	0.800
Catboost	0.787	0.849	0.636	0.810
Logistic Regression	0.800	0.811	0.773	0.864

### Feature importance analysis

**Figure 3:**
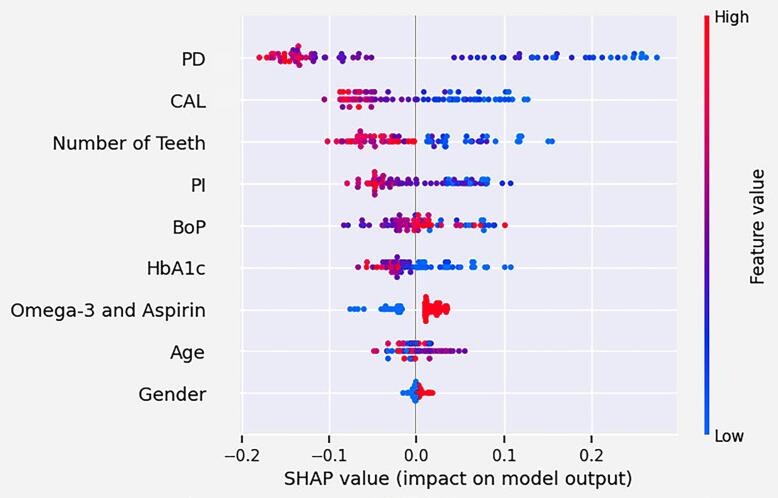
The SHAP value summary plot shows features that contribute to the prediction model. The features are ordered according to their importance to the model. The red color corresponds to a high impact on the prediction model, while the blue color corresponds to a low impact. “Gender” refers to female.

The SHAP value plot, illustrated in Figure 3, enables us to evaluate which features had a more significant influence on the outcome of the RF model. Nine variables were included in the algorithm, with the mean PD and mean CAL being the most important contributors to the model. Regarding the mean PD, mean CAL, number of teeth, PI, and HbA1c, higher values are associated with a reduced contribution to the prediction of achieving the endpoint in periodontal treatment, while lower values indicate greater impact. On the other hand, lower values of adjunct omega-3 and aspirin (receiving placebo) indicate a reduced likelihood of achieving the endpoint.

## Discussion

By combining the baseline periodontal, metabolic, and demographic parameters from patients with periodontitis and type 2 DM, we built an AI model that predicts responses to periodontal treatment. The model presented an accuracy of 80%, a specificity of 87%, and an AUC of 0.84. We observed that the combination of nine factors (out of the 31 initially evaluated) could help identify whether patients achieved the clinical endpoint for periodontal treatment or not. Our model offers a tangible framework to understand the relative importance of each parameter. By providing a hierarchical perspective, the model serves as a practical tool to guide clinicians and patients in understanding the significance of each variable for treatment outcomes.

This study aimed to predict periodontal treatment outcomes in patients with periodontitis and type 2 DM using AI models, namely ML techniques, considering periodontal parameters, metabolic status, and demographic characteristics at baseline. To this end, we defined the clinical endpoint for treatment as the primary outcome of the study (≤4 sites with PD ≥5 mm). This is a binary variable based on a treat-to-target outcome. A recent meta-analysis that compared several proposed endpoints for periodontal treatment showed a significant association between not achieving this endpoint and tooth loss within at least five years of supportive periodontal care (relative risk = 2.57; p = 0.003).^16^ Thus, the rationale for identifying factors that influence the response to treatment was based on an endpoint that has been considered statistically accurate and clinically relevant.^12,16^

To identify such features, we used AI technology to train a clinical dataset using a double-blind placebo-controlled RCT that evaluated the effects of omega-3 and aspirin as adjuncts to periodontal debridement in the treatment of periodontitis in patients with type 2 DM.^11^ Via data analysis, we identified baseline clinical, metabolic, and demographic factors that could indicate whether a patient would achieve the target for periodontal therapy. These findings may help clinicians identify profiles of good and poor responders in the decision-making process of treatment planning. Nevertheless, AI models should be regarded as additional tools in clinical decision-making, and not as a definite prognostic marker in patient care.

The factors that contributed most to the AI model included clinical, metabolic, and demographic variables and the use of adjunctive therapy. Full mouth mean PD emerged as the most influential factor affecting the likelihood of achieving the treatment endpoint. This finding reinforces the recommendation to consider PD as a fundamental variable for assessing reduced periodontium health in clinical practice and reassessing clinical parameters after subgingival instrumentation.^15-18^

Mean CAL was the second most relevant factor in the algorithm, with lower baseline levels indicating a greater chance of achieving the treatment endpoint. The diagnosis of periodontitis progression relies on changes in CAL over time. A classification study using linear mixed models based on CAL to identify different patterns of disease progression at the site and patient level showed that patients with severe forms of periodontitis were more prone to present active disease progression than healthy and mild periodontitis patients.^19^ Similarly, we identified patients presenting mean CAL > 4.0mm at baseline that, especially when correlated with higher levels of HbA1c (> 9.4%), were less likely to achieve the target of treatment. These altered patterns in severe periodontitis may be explained by inherent immunoinflammatory dysregulation towards dysbiosis, which appears to be enhanced in individuals with uncontrolled DM.

PI was the third factor with the most relevant contribution to the model. In periodontal trials, PI is a surrogate outcome measured as an attempt to show how oral hygiene is managed during the study within treatment groups, but little is known about the impact of biofilm accumulation on treatment response at the individual level. Comparing means of surrogate variables cannot show particular characteristics that influence treatment results. In the original RCT, the patients received personalized oral hygiene instructions and supragingival biofilm removal before subgingival instrumentation followed by monthly supragingival biofilm control. However, the biofilm accumulation profile before the first step of periodontal therapy greatly influenced the treatment results after six months, even though the mean PI decreased over time for all treatment groups.

It is well-known that biofilm accumulation is the etiology of gingivitis and that supragingival biofilm control by the patient is essential to obtain periodontal stability.^20,21^ The accumulation of biofilm initiates inflammatory and immune responses that lead to the pathogenesis of periodontitis.^22^ Due to trained immunity, patients with severe periodontitis and uncontrolled DM presented exacerbated immune responses to bacteremia and systemic inflammation, resulting in a faster and stronger response to low levels of cytokines and bacterial toxins, culminating in an insufficient response to periodontal treatment.^23^

Our findings also highlight the need for future studies aimed at treating patients who did not respond well to treatment. Patients that presented HbA1c >9.4%, with mean PD >3.5 mm, CAL >4.0 mm, and >40 sites with PD ≥5mm at baseline formed clusters in the opposite direction of the good responders. These findings emphasize the need for new studies that focus on highly uncontrolled DM patients, which represents a challenge from the dental and medical perspectives. A multidisciplinary approach that includes behavioral changes and pharmacological treatments to improve metabolic control, together with periodontal treatment and prevention, should be implemented as a global effort to prevent and delay the risk for complications and promote quality of life for people living with DM.^24^

The level of hyperglycemia, rather than the diagnosis and etiology of DM, is associated with periodontitis and the probability of tooth loss.^5,25^ Although the threshold for uncontrolled DM associated with periodontitis has not yet been established—with HbA1c levels ranging from 7% through 9% in epidemiological studies—uncontrolled DM seems to impact periodontitis progression and tooth loss.^26-28^ A recent study showed that patients with uncontrolled DM have a high risk of periodontitis recurrence after 4–5 years, even if treated with systemic metronidazole and amoxicillin.^29^ When comparing people with and without DM regarding periodontal therapy, short-term scaling and root planing (SRP) results seem not to be affected by glycemic control.^28^ However, most studies comparing the two populations have a 3-month follow-up, which, combined with the significant variations in the threshold for uncontrolled DM, impairs clinically meaningful considerations. Thus, future studies should investigate the outcomes of periodontal therapy and the variables that influence those outcomes for uncontrolled DM patients in the long term.

The ML model corroborates the results of the original RCT and other previous RCTs and meta-analyses that demonstrated the effectiveness of adjunctive omega-3 in treating periodontitis.^30-35^ Using AI approaches, we also observed that replacing omega-3 and aspirin with a placebo lowered the likelihood of achieving the endpoint for treatment. As reported in the RCT, the groups that received omega-3 and aspirin had a higher percentage of patients achieving the endpoint for periodontal treatment when compared to the control group. In future RCTs, incorporating AI methods could serve as supplementary tools for evaluating the efficacy of treatment outcomes. This would involve considering both the impact of the treatment being studied and the factors under examination.

In general, the accuracy of 80%, the specificity of 87%, and the AUC of 84% obtained using the Random Forest model are comparable to the results reported in systematic reviews in the medical and dental fields, in which clinical interventions have been assessed.^36-38^ However, further improvements and validation may still be desirable to enhance the reliability and effectiveness of the model for real-world clinical use. Thus, there are some limitations to implement this model in routine clinical practice. Firstly, the algorithm should be applied to clinical cases with similar characteristics to our study population. Secondly, the model may only be applied to patients receiving the same treatment protocols from the RCT (periodontal debridement associated or not with omega-3 and aspirin). For different treatment modalities, such as the use of other adjunctive therapies, new ML models should be built, tested, and evaluated. Nevertheless, increasing the sample size of the cohort and incorporating additional features into the model may improve the predictive performance and its generalizability.

Of note, the specificity of 87% supports the effectiveness of the model in predicting negative outcomes. By identifying the factors that could influence negative outcomes, clinicians may use the ML model as an adjunctive decision-making tool because of the increased probability that the patient will need step 3 periodontal therapy. Additionally, researchers can design new studies that focus on these factors and patient profiles. This approach would contribute to building evidence regarding the previously unobserved unsuccessful clinical trial scenarios and open new avenues for study populations.

The developed model can serve as a valuable adjunctive tool, addressing uncertainties surrounding the interpretation and application of findings from RCTs, particularly concerning factors that influence the response to periodontal therapy in patients with uncontrolled DM. Beyond its clinical application, the model holds significant promise in patient education concerning oral health. By emphasizing the importance of behavioral changes regarding oral hygiene—such as reducing biofilm accumulation, and managing risk factors, like DM control—the model aligns with the EFP guidelines for the treatment of stages of I to III periodontitis.^18^ This focus on early (step 1) intervention underscores its potential to yield more favorable treatment outcomes. Notably, by facilitating improved oral health practices and addressing risk factors, the model has the potential to not only enhance treatment success but also mitigate overall treatment costs, thereby advancing oral healthcare on a broader scale.

Furthermore, in clinical periodontics, predictive AI models can support personalized decision-making by assisting clinicians in identifying patients at higher risk of poor response to therapy and tailoring follow-up strategies accordingly. These models can inform the frequency and intensity of supportive periodontal care, aid in setting realistic treatment expectations, and support shared decision-making processes with patients. As data integration and model refinement advance, these tools could be incorporated into chairside decision-support systems, enhancing the precision and efficiency of periodontal care delivery.

In parallel with these advancements, it is equally important to revisit how we define treatment success in periodontics, particularly when managing patients with chronic systemic conditions. The concept of periodontal treatment success warrants a more nuanced interpretation, particularly in the context of chronic, multifactorial conditions such as DM and periodontitis. Traditional definitions often rely on the attainment of standardized clinical thresholds, yet such metrics may not fully capture the therapeutic benefit experienced by patients with a high baseline disease burden. Similar to diabetes management, in which improvements in glycemic control are considered meaningful even if target HbA1c levels are not fully achieved, periodontal therapy should also be evaluated through the lens of individual progress. For patients with uncontrolled DM and advanced periodontitis, substantial improvements in clinical parameters, inflammation reduction, or symptom relief may represent a significant positive shift in overall health and quality of life. This perspective emphasizes the value of treatment even among those traditionally labeled as “poor responders,” recognizing that success can be defined by the degree of improvement and its relevance to the patient’s condition. Embracing this broader, more individualized view aligns with a patient-centered approach to chronic disease management and reinforces the potential role of predictive models in supporting tailored therapeutic strategies and realistic expectations.

## Conclusion

The combination of nine baseline periodontal, metabolic, and demographic factors from patients with periodontitis and type 2 DM may help predict the response to periodontal therapy. Lower baseline levels of full mouth mean PD, mean CAL, plaque index, and HbA1c increased the likelihood of achieving the treatment endpoint at the 6-month follow-up. However, all nine features included in the model should be considered for treatment outcome predictability. Clinicians may consider the characterization of periodontal therapy responses to implement personalized care and treatment decision-making.
